# Monocytes enhance the inflammatory response to TLR2 stimulation in aortic valve interstitial cells through paracrine up-regulation of TLR2 level

**DOI:** 10.7150/ijbs.49332

**Published:** 2020-10-03

**Authors:** Peijian Zhang, Erlinda The, Balachandar Nedumaran, Lihua Ao, Michael J. Jarrett, Dingli Xu, David A. Fullerton, Xianzhong Meng

**Affiliations:** 1 Department of Surgery, University of Colorado Denver, Aurora, CO 80045.; 2 Department of Cardiology, Nanfang Hospital, Southern Medical University, Guangzhou, China.

**Keywords:** Aortic valves, inflammation, adhesion molecules, monocytes, Toll-like receptor

## Abstract

**Background and Objectives:** Chronic valvular inflammation associated with monocyte infiltration promotes calcific aortic valve disease (CAVD) progression. Further, innate immunity in aortic valve interstitial cells (AVICs), mediated by Toll-like receptors (TLRs), up-regulates cellular inflammatory, fibrogenic and osteogenic activities. Currently, the pro-inflammatory communication between monocytes and AVICs and the underlying mechanism are unclear. We hypothesized that monocytes up-regulate AVIC inflammatory activity. This study sought to characterize the interaction between monocytes and AVICs and to elucidate the mechanism underlying cell-to-cell communication.

**Methods and Results:** AVICs, monocytes and co-cultures were exposed to a low concentration of TLR2 activator Pam3CSK4 (0.03 µg/ml). The TLR2 activator at this dose induced a marked increase in AVIC production of ICAM-1 and VCAM-1 only when co-cultured with monocytes. Adding conditioned medium from Pam3CSK4-treated monocytes (Pam3 CM, containing 0.1 µg/ml of Pam3CSK4) to AVIC culture (30% vol/vol; diluting Pam3CSK4 to 0.03 µg/ml) greatly increased the expression of adhesion molecules while adding conditioned medium from untreated monocytes (control CM) had no effect. Inhibition or knockdown of TLR2 in AVICs markedly reduced ICAM-1 and VCAM-1 expression induced by Pam3 CM. Further, Pam3 CM increased TLR2 levels in AVICs. Multiplex-ELISA analysis of Pam3 CM identified greater levels of TNF-α. Neutralization of TNF-α abolished the effect of Pam3 CM on AVIC TLR2 levels, resulting in marked attenuation of its potency in the induction of adhesion molecule expression.

**Conclusions:** This study demonstrates that activated monocytes use paracrine signaling to sensitize AVICs for inflammatory responses to a low level of TLR2 activator. The mechanism of sensitization involves up-regulation of AVIC TLR2 levels by TNF-α from monocytes. Infiltrated monocytes in aortic valve tissue may exacerbate valvular inflammation by rendering AVICs hypersensitive to TLR2 activators.

## Introduction

Calcific aortic valve disease (CAVD) is one of the leading cardiovascular diseases in the elderly 1. This disease is a chronic inflammatory condition, and progressive fibrosis and calcification of inflammatory valve leaflets eventually result in aortic stenosis and heart failure 2-5. CAVD progression to severe aortic stenosis takes several years. While this observation indicates that there is a wide therapeutic window for prevention of CAVD progression, no effective pharmacological intervention is available for the prevention. Currently, the only available therapy is surgical or transcatheter aortic valve replacement to treat patients with severe aortic stenosis.

The predominant cells in human aortic valve leaflets are the aortic valve interstitial cells (AVICs). Accumulating evidence supports the notion that AVICs are important players in valvular inflammation and CAVD progression 6. In this regard, numerous studies including ours demonstrate that human AVICs have pro-inflammatory potential 7-9. Moreover, we observed that human AVICs express functional Toll-like receptors (TLRs), and AVICs isolated from diseased aortic valves have higher levels of TLR2 and TLR4 10. More importantly, stimulation of TLRs in human AVICs not only induces inflammatory responses 7-9, 11, but also elevates cellular osteogenic and fibrogenic activities 8-10, 12-14, and the pro-inflammatory and pro-osteogenic pathways in human AVICs interact to modulate cellular inflammatory and osteogenic activities 15. Thus, TLRs may play a mechanistic role in mediating CAVD progression 16. It is reasonable to postulate that pro-inflammatory activation of AVICs promotes CAVD progression. Further work in this area may identify therapeutic targets for suppression of CAVD progression through modulation of AVIC inflammatory activity.

Monocyte infiltration and monocyte/macrophage accumulation in aortic valves are characteristic pathobiology of progressive CAVD 17-19, and M1 macrophages have been found to be associated with congenital bicuspid aortic valve stenosis 20. While monocytes are recognized as important immune effector cells in promoting vascular inflammation 21, their influence on the overall valvular inflammation associated with CAVD progression remains to be evaluated. It is known that infiltrated monocytes are able to affect nearby cells through direct cell-cell interaction (juxtacrine) and/or indirect interaction by releasing pro-inflammatory factors (paracrine) 22, 23. To date, limited studies on the interaction of monocytes/macrophages with AVICs have been reported. In this regard, murine monocytes were found to promote human AVIC osteogenic activity by a mechanism involving the miR-214/TWIST1 pathway 24. In addition, a previous study found that conditioned medium from activated human monocytes/macrophages increases human AVIC osteogenic activity if AVICs are cultured in a pro-calcification condition, indicating that activated monocytes/macrophages enhance AVIC response to a pro-osteogenic environment 18. Currently, the impact of activated monocytes/macrophages on AVIC inflammatory activity is unclear.

We hypothesized that activated monocytes enhance AVIC inflammatory response to TLR stimulation. The purpose of the present study was to investigate pro-inflammatory communication of activated monocytes with AVICs and the mechanism by which monocytes modulate the inflammatory activity in AVICs.

## Materials and Methods

### Chemical and reagents

Antibody against ICAM-1 (sc-7891) was obtained from Santa Cruz Biotechnology, Inc. (Santa Cruz, CA). Antibodies against VCAM-1 (E1E8X) and MyD88 (D80F5) were obtained from Cell Signaling, Inc. (Beverly, MA). Antibody against TLR2 (NB100-56728) was obtained from Novus Biologicals, LLC (Centennial, CO). Antibodies against β-actin (ab49900) and GAPDH (ab9385) were obtained from Abcam (Cambridge, MA). Antibody against vimentin (ab137321) was obtained from Abcam (Cambridge, MA). Medium 199 (M199) was obtained from Lonza (Walkersville, MD). Roswell Park Memorial Institute medium (RPMI 1640) was obtained from GIBCO Laboratories (Grand Island, NY). Pam3CSK4 was obtained from InvivoGen (San Diego, CA). TLR2 inhibitor CU CPT 22 (4884) was obtained from Tocris Bioscience (Bristol, UK). Tumor necrosis factor-α (TNF-α) neutralizing antibody (MAB 610) was purchased from R&D System (Minneapolis, MN). Recombinant TNF-α, collagenase and other reagents were obtained from Sigma-Aldrich Chemical Co. (St Louis, MO).

### Isolation and culture of human primary AVICs

Normal aortic valves were collected from explanted hearts of patients having cardiomyopathy and undergoing heart transplantation. The valve leaflets were thin and did not have histological abnormality. This study was approved by the University of Colorado Multiple Institution Review Board (IRB Protocol 08-0280). All subjects gave their informed consent for the use of their aortic valves for this study. The investigations were carried out following the rule of the Declaration of Helsinki of 1975, revised in 2013.

AVICs were isolated and cultured using a previously described method 25. Briefly, valve leaflets were subjected to sequential digestions with collagenase. A high concentration of collagenase (2.5 mg/mL) was used to remove endothelial cells. Then, the tissue was digested in a solution containing 0.8 mg/mL of collagenase to free the interstitial cells, and AVICs in the solution were harvested by centrifugation. Cells were maintained in M199 growth medium supplemented with 10% fetal bovine serum, penicillin G (100 units/mL), streptomycin (100 mg/mL) and amphotericin B (0.25 µg/mL). Cells of passage 3 to 6 were used for the experiments when the cultures reached approximately 80% confluence.

### Culture of THP-1 monocyte

THP-1 monocyte cell line was purchased from American Type Culture Collection (Manassas, VA). According to manufacturer's instruction, THP-1 cells were cultured in T-75 flask with RPMI 1640 growth medium supplemented with 10% fetal bovine serum in an incubator with 5% CO2 at 37 °C. The medium was renewed every 2-3 days. Cells were subcultured when cell density was approximately 8-10 ×10^5^ cells/ml.

Cells cultured in 24-well plates were treated with Pam3CSK4 at 0.03 μg/ml for 24 hours or left untreated. After washing, cells were collected for examining levels of ICAM-1 and VCAM-1.

### Co-culture of AVICs and monocytes

In the co-culture system, monocytes and AVICs are cultured together and paracrine and juxtacrine signaling may be involved in the interaction between these cells. To perform AVIC-monocyte co-culture, monocytes were collected from culture medium when their concentration approximated 8-10 ×10^5^ cells/ml. For immunoblotting analysis, 3×10^4^ of AVICs and 1×10^5^ of monocytes were co-cultured in each well of a 24-well plate, and 1×10^4^ of AVICs and 3×10^4^ of monocytes were seeded onto each chamber of an 8-chamber slide for immunofluorescence analysis. AVICs and monocytes were co-cultured overnight in RPMI 1640 growth medium with 2.5% fetal bovine serum before experiment.

Co-cultures were treated with Pam3CSK4 at 0.03 μg/ml for 24 hours or left untreated. After washing, cells were collected for examining levels of ICAM-1 and VCAM-1. Monocyte cultures and AVIC cultures were treated in the same manner to serve as controls.

### Treatment of AVICs with conditioned medium

To determine monocyte paracrine effect, conditioned medium from monocyte culture is applied to a different cell type in a separate culture. This eliminates the effect of juxtacrine signaling from monocytes 26, 27. For this purpose, we collected conditioned media from monocyte cultures that were untreated or treated with Pam3CSK4 at 0.1 μg/mL for 24 hours.

To generate conditioned medium, monocytes were seeded in 12-well plates in a density of 2×10^5^ cells/ml and incubated in medium containing 2.5% fetal bovine serum overnight before treatment. Monocytes were treated with Pam3CSK4 at 0.1 μg/mL for 24 hours or left untreated. Culture media were collected after treatment. The supernatant from Pam3CSK4-treated monocyte culture is described as Pam3CSK4 conditioned medium (Pam3 CM). The supernatant from untreated monocyte culture is described as control conditioned medium (control CM).

Conditioned medium was stored in -80 °C before use. For the experiment, a mixture of 0.15 mL conditioned medium and 0.35 mL growth medium [30% (vol/vol) of conditioned medium] was applied to AVICs in 24 well plate.

To knockdown TLR2 in AVICs, cells were transduced with lentivirus expressing control shRNA or TLR2 shRNA following polybrene treatment. Five days later, the efficiency of TLR2 knockdown was validated by immunoblotting. To inhibit TLR2 in AVICs, specific TLR2 inhibitor CU CPT 22 (10 µM) was applied to AVICs 1 h prior to subsequent experiment.

To neutralize TNF-α in the Pam3 CM, the CM was treated with TNF-α neutralizing antibody at 10 µg/ml for 1 h at 37 °C prior to applying the CM to AVIC culture. Aliquots of the CM were treated with non-immune IgG and used as controls.

### Immunoblotting

Immunoblotting was performed to assess levels of ICAM-1, VCAM-1, TLR2 and MyD88 in AVIC lysate. AVICs were washed with PBS and lysed in 2× Laemmli sample buffer (Bio-Rad Laboratories, Inc., Hercules, CA). AVICs of 2 wells under same experimental condition from 24-well plate were pooled as one sample. Cell lysate samples were separated on 4-20% SDS-PAGE gels and transferred to nitrocellulose membranes. The membranes were blocked with 5% skim milk solution for 1 hour at room temperature, incubated with primary antibodies (1:200 to 1:1000 volume dilutions) overnight at 4 °C, and then incubated with secondary antibodies for 2 hours at room temperature. Membranes were developed using the enhanced chemiluminescence system. The levels of detected proteins were normalized against GAPDH or β-actin. Densitometric analysis of protein band was performed using the ImageLab software (Bio-Rad Laboratories, Inc., Hercules, CA).

### Immunofluorescence staining

Immunofluorescence staining was performed to detect ICAM-1 and vimentin in co-cultured monocytes and AVICs. Monocytes and AVICs were co-cultured in 8-well chamber slides and left untreated or treated with Pam3CSK4 (0.03 μg/ml) for 24 hours. To detect TLR2 in human AVICs, cells (1×10^4^ cells/well) were cultured in 8-well chamber slides (with 210 µL of culture medium) and treated with Pam3 CM (90 µl/well) for 4, 8, 12 and 24 hours. After treatment, cells were rinsed gently with PBS and fixed with 4% paraformaldehyde for 10 minutes at room temperature. Non-specific binding of antibodies were blocked with 10% goat serum for 30 minutes at room temperature. Co-cultured monocytes and AVICs with or without Pam3CSK4 treatment were incubated with antibodies against ICAM-1 and vimentin at 4 °C overnight. AVICs exposed to Pam3 CM were incubated with an antibody against TLR2. Following washed with PBS, cells were incubated with Alexa 488 (Green) or Cy3 (red)-conjugated secondary antibody for 2 hours at room temperature. DAPI was used for nuclear counterstaining. Cells were analyzed and imaged using a Leica CTR5500 digital microscope (Leica Mikroskopie und Systeme GmbH, Wetzlar, Germany).

### ELISA

Multiplex ELISA arrays (RayBiotech, Inc., Peachtree Corners, GA) were utilized to quantify levels of GM-CSF, G-CSF, TNF-α, IL-1α, IL-1β, IFN-γ, IL-6, IL-8, IL-11, IL-17, TGF-β1, MCP-1, BMP-2, BMP-7, VEGF, MIP-1α, RANKL and RANTES in the conditioned medium. Samples and standards were prepared following manufacturer's instructions. Scanning and data extraction were performed by RayBiotech, Inc. By comparing signals of samples to the standard curve, cytokines concentrations in samples were determined and results plotted using Prism Software (GraphPad).

Single cytokine ELISA kits (R&D Systems, Minneapolis, MN) were used to analyze MCP-1 levels in supernatant from AVICs culture and to validate the data of TNF-α from multiplex ELISA assay. Samples and standards were prepared according to manufacturer's instructions. Absorbance of standards and samples were determined spectrophotometrically at 450 nm, using a microplate reader (Biotek, Winooski, VT). Results were plotted against the linear portion of the standard curve.

### Statistical analysis

Statistical analyses were performed using Prism Software (GraphPad). Data are presented as mean ± SEM. ANOVA with the post hoc Fisher test was performed to analyze differences between multiple groups, and *t*-test was applied to compare data between two groups. Nonparametric Mann-Whitney U test was performed to confirm the difference of two group comparison. For multiple group comparisons, nonparametric Kruskal-Wallis test was performed to confirm the differences. Statistical significance was defined as *P*≤0.05.

## Results

### Monocyte and AVIC co-culture display enhanced inflammatory responses to TLR2 stimulation

**Figure 1:**
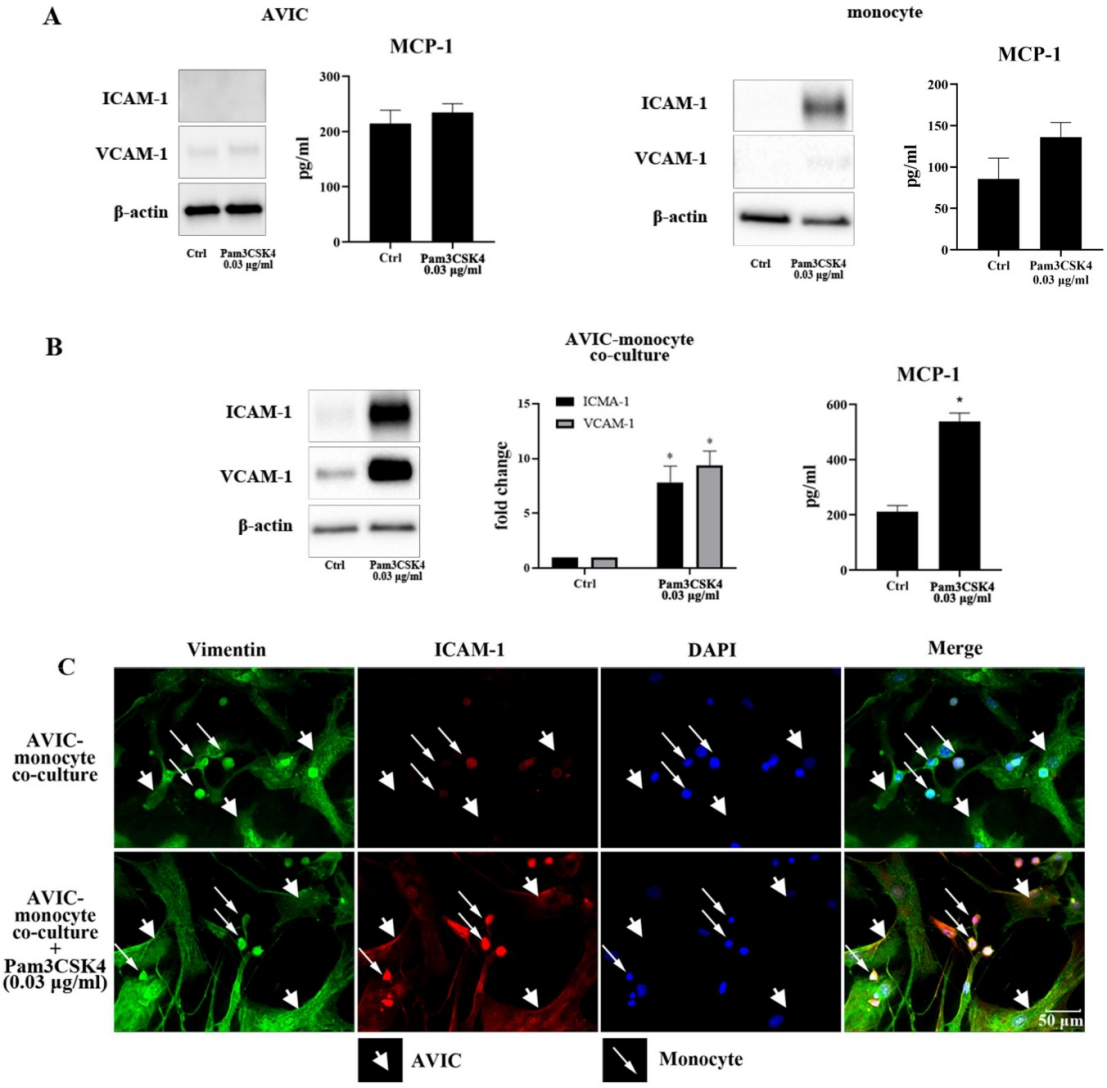
** Pam3CSK4 induces inflammatory responses in AVIC and monocyte co-culture. A.** AVICs and monocytes were cultured separately and treated with a low dose of Pam3CSK4 (0.03 µg/ml) for 24 h. Representative immunoblots and ELISA data of 4 separate experiments show that Pam3CSK4 elevates ICAM-1 level moderately and causes a slight increase in MCP-1 production in monocytes while Pam3CSK4 in this low dose has no effect on the levels of ICAM-1, VCAM-1 and MCP-1 in AVICs. **B.** AVIC and monocyte co-cultures were treated with Pam3CSK4 (0.03 µg/ml) for 24 hours or left untreated. Representative immunoblots and ELISA data show that low levels of ICAM-1, VCAM-1 and MCP-1 are present in untreated control. However, stimulation of TLR2 with a low dose of Pam3CSK4 markedly up-regulates the production of ICAM-1, VCAM-1 and MCP-1 in co-cultures. Data are presented as mean ± SEM. n=4 cell isolates from distinct donor valves, *P<0.05 vs. control. **C.** Representative images of ICAM-1 immunofluorescence staining from 3 experiments show that both AVICs and monocytes express ICAM-1 (red) following the exposure to a low dose of Pam3CSK4 (0.03 µg/ml). Immunofluorescence staining of vimentin (green) and DAPI counterstaining (blue) were applied to identify AVICs and to outline all nuclei in the co-culture. Original magnification 40× objective.

As shown in **Figure 1A**, stimulation with Pam3CSK4 (0.03 μg/ml) did not increase ICAM-1, VCAM-1 or MCP-1 in AVICs, but moderately increased ICAM-1 level and MCP-1 secretion in monocytes. Although Pam3CSK4 in this concentration enhanced MCP-1 secretion in monocytes, the difference from control level was not statistically significant. ICAM-1, VCAM-1 and MCP-1 levels were markedly up-regulated when monocytes and AVICs were co-cultured and exposed to this low concentration of Pam3CSK4 (**Figure 1B**). Immunofluorescence staining confirmed that Pam3CSK4-induced a significant increase in ICAM-1 expression in the co-culture, and this adhesion molecule was localized in both monocytes and AVICs (**Figure 1C**). The observation suggests that activated monocytes enhance AVIC inflammatory response to TLR2 stimulation.

### Pam3 CM induces the inflammatory response in AVICs in a TLR2-dependent fashion

**Figure 2:**
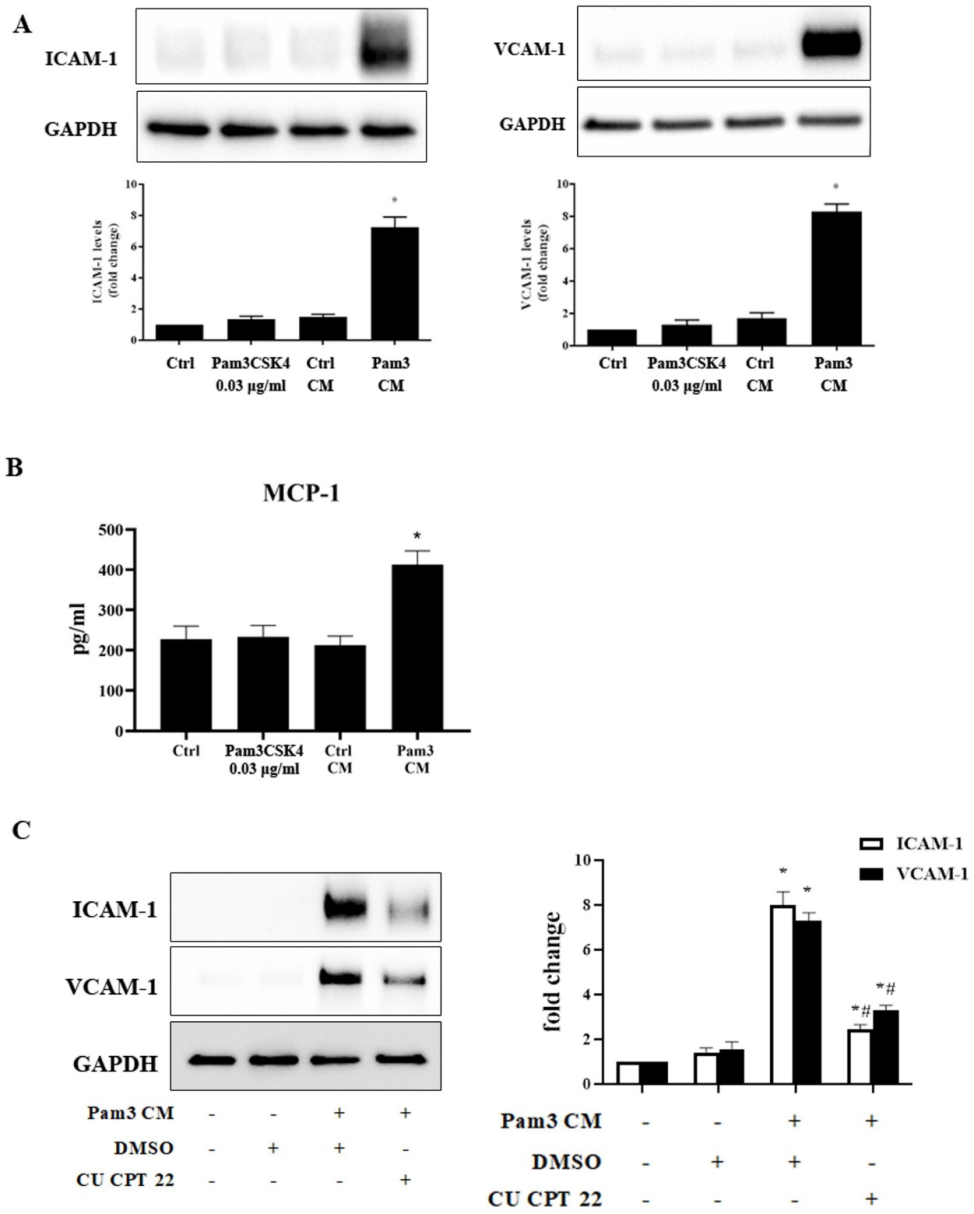
** Conditioned medium from Pam3CSK4-treated monocytes induces inflammatory responses in human AVICs.** Conditioned medium was collected from monocyte cultures treated with Pam3CSK4 (0.10 µg/ml, Pam3 CM) or untreated controls (control CM). Pam3 CM and control CM, 0.15 ml each, were added to AVIC culture in 0.35 ml growth medium [achieving a 30% (vol/vol) for CM in AVIC culture], and AVICs were treated with control CM or Pam3 CM for 24 hours. **A.** Representative immunoblots and densitometric data show that Pam3 CM markedly up-regulates ICAM-1 and VCAM-1 expression in AVICs while neither control CM nor 0.03 µg/ml of Pam3CSK4 (equivalent to the concentration in Pam3 CM-treated AVIC culture) demonstrated this effect. *P<0.05 vs. untreated control**. B.** ELISA data shows that Pam3 CM stimulates AVICs to produce and release MCP-1. *P<0.05 vs. untreated control.** C.** AVICs were treated with DMSO or CU CPT 22 for 1 hour before Pam3 CM treatment for 24 hours. Inhibition of TLR2 in AVICs markedly attenuated ICAM-1 and VCAM-1 expression induced by Pam3 CM. *P<0.05 vs. untreated control, *#P*<0.05 vs. Pam3 CM+DMSO. All data are presented as mean ± SEM. n=4 cell isolates from distinct donor valves.

As monocytes can interact with neighboring cells via a paracrine mechanism 28, we tested the hypothesis that activated monocytes secrete soluble factors to sensitize AVICs to respond to a low level of TLR2 activator for inflammatory responses via a paracrine effect. Conditioned medium derived from untreated monocytes (control CM) and Pam3CSK4-treated monocytes (Pam3 CM) were applied to treat AVICs. Immunoblotting revealed that cellular ICAM-1 and VCAM-1 levels increase in a time-dependent fashion in human AVICs (**Figure S1**) following an exposure to Pam3 CM (Pam3CSK4 concentration becoming 0.03 μg/ml after dilution by the medium in AVIC culture). Further, levels of these adhesion molecules peaked at 24 hours (**Figure S1 and Figure 2A**). ELISA analysis identified the secretion of inflammatory cytokines by AVICs after an exposure to Pam3 CM. As shown in **Figure 2B**, MCP-1 level in culture medium was markedly increased following treatment of AVICs with Pam3 CM. However, control CM and Pam3CSK4 alone (0.03 μg/ml) had no effect (**Figures 2A and 2B**). The results indicate that activated monocytes exert a paracrine effect on AVICs to enhance their sensitivity to TLR2 stimulation by a sub-threshold level of activator.

To test the hypothesis that enhanced response to TLR2 stimulation is responsible for the up-regulation of ICAM- and VCAM-1, we examined whether inhibition of TLR2 affects Pam3 CM-induced expression of ICAM-1 and VCAM-1 by applying the specific TLR2 inhibitor CU CPT 22 to AVICs prior to Pam3 CM treatment. As shown in **Figure 2C**, inhibition of TLR2 in AVICs significantly reduced the expression of ICAM-1 and VCAM-1. Thus, AVICs displayed TLR2 response to Pam3 CM that carries 0.03 μg/ml of Pam3CSK4 to the AVIC culture although AVICs did not respond to 0.03 μg/ml of Pam3CSK4 added to the culture medium. It appears that factors released by activated monocytes sensitize AVICs for the response to a low level of TLR2 activator.

### Conditioned medium from activated monocytes up-regulates TLR2 levels in AVICs

**Figure 3:**
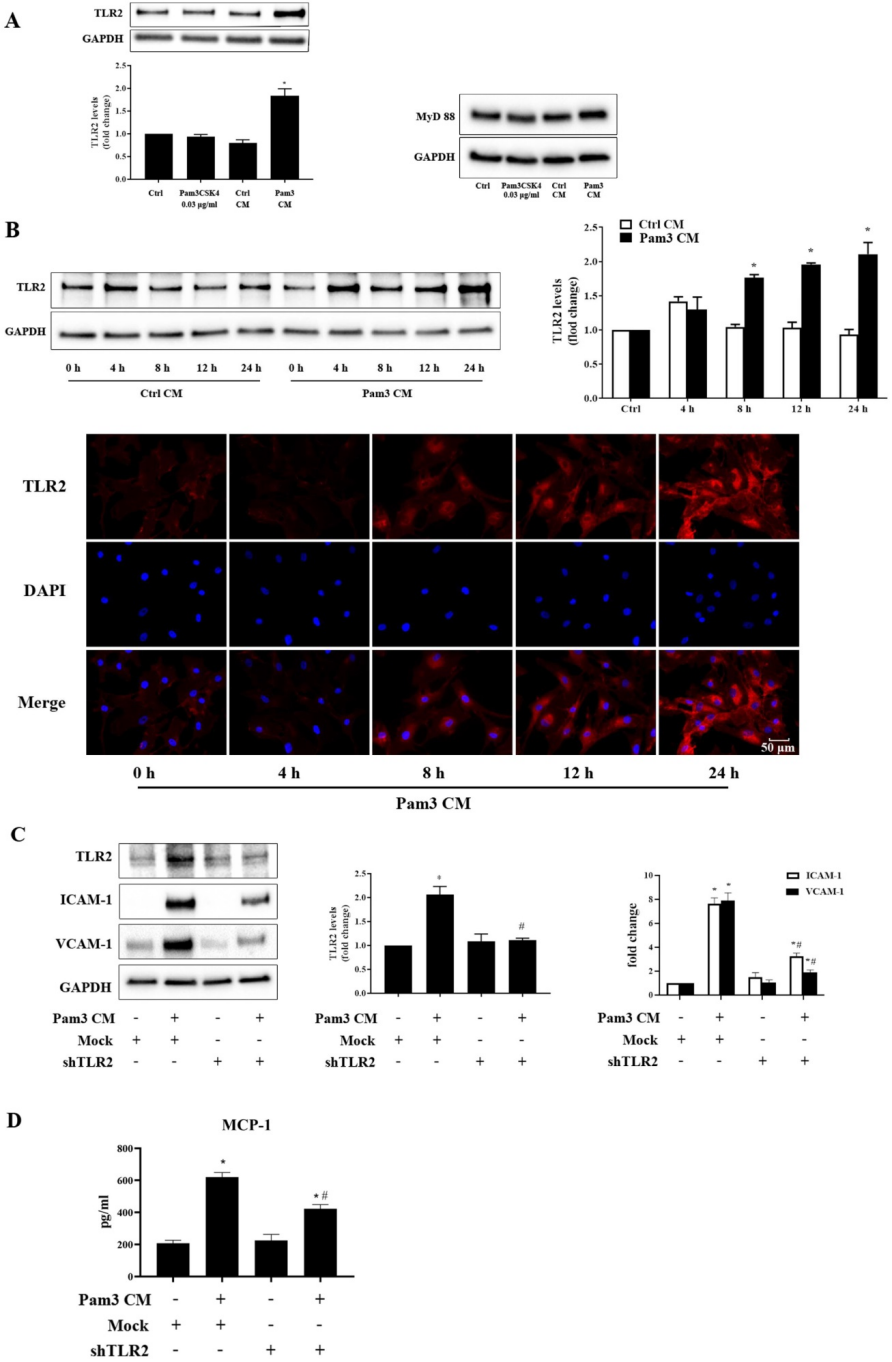
** Pam3 CM up-regulates TLR2 expression in AVICs to elicit cellular inflammatory responses. A.** AVICs were exposed to Pam3CSK4 (0.03 μg/ml), control CM (30%) or Pam3 CM (30%) for 24 hours. Representative immunoblots and densitometric data show that Pam3 CM elevates TLR2 levels, but not MyD88 levels in AVICs. *P<0.05 vs. untreated control, Pam3CKS4-treated and control CM-treated. **B.** AVICs were treated with control CM or Pam3 CM for 4 to 24 hours**.** A representative immunoblot and densitometric data show that cellular TLR2 levels increase at 8, 12 and 24 hours following the exposure to Pam3 CM. *P<0.05 vs. control CM treatment for the same time period. Representative images of immunofluorescence staining confirmed the up-regulation of TLR2 (red) at 8-24 hours of treatment with Pam3 CM. The nuclei were visualized with DAPI (blue) counterstaining. Original magnification 40× objective. **C and D.** AVICs were transduced with lentivirus expressing control (mock) shRNA or TLR2 shRNA. After expression for 5 days, cells were treated with Pam3 CM for 24 hours. Knockdown of TLR2 abolished TLR2 up-regulation induced by Pam3 CM, and reduced the production of adhesion molecules and MCP-1. *P<0.05 vs. mock transduction, *#P*<0.05 vs. Pam3 CM + mock transduction. All quantitative data are presented as mean ± SEM. n=4 cell isolates from distinct donor valves in each group.

To understand whether factors from activated monocytes enhance AVIC response to TLR2 stimulation by increasing TLR2 levels, we evaluated the effect of Pam3 CM on TLR2 levels in AVICs. As shown in **Figure 3A**, Pam3 CM increased TLR2 levels in AVICs without an influence on MyD88 levels. The time trial data showed that TLR2 levels in AVICs are higher at 8 hours after being exposed to Pam3 CM and further increase from 8 to 24 hours (**Figure 3B**). The results of immunofluorescence staining confirmed that an exposure of AVICs to Pam3 CM increased TLR2 levels at 8-24 hours (**Figure 3B**). It appears that higher levels of TLR2 in AVICs are involved in enhancing their inflammatory response. Indeed, knockdown of TLR2 using lentiviral shRNA abrogated the increase in TLR2 level and markedly reduced the up-regulation of ICAM-1 and VCAM-1, and the production of MCP-1 (**Figure 3C and 3D**). Thus, factors from activated monocytes elevate TLR2 levels in AVICs, and high levels of TLR2 play a major role in over-expression of adhesion molecules in AVICs exposed to the Pam3 CM.

### TNF-α is responsible for up-regulation of TLR2 and enhancement of inflammatory response in AVICs

**Figure 4:**
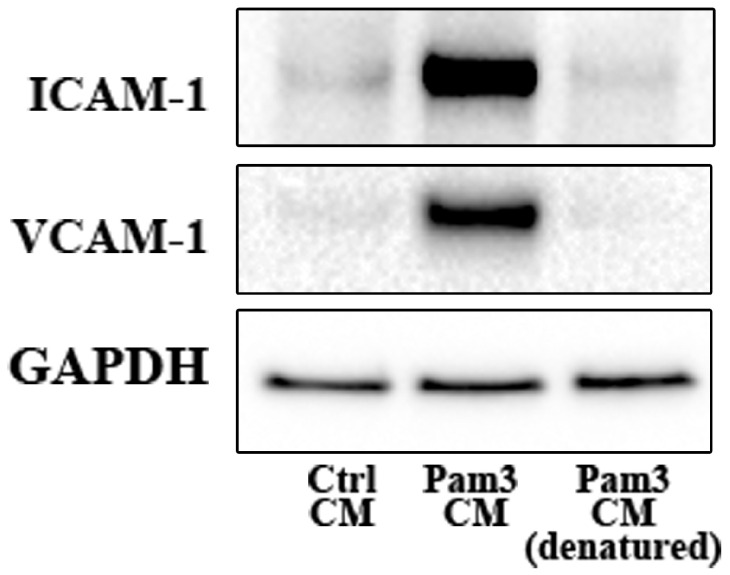
** Denatured Pam3 CM fails to induce AVIC inflammatory responses.** AVICs were treated with control CM, Pam3 CM or heat-denatured Pam3 CM for 24 hours. A representative immunoblot of 3 experiments shows that denatured Pam3 CM lost its effect on the expression of ICAM-1 and VCAM-1 in AVICs.

We tested the hypothesis that pro-inflammatory mediators released by activated monocytes are the paracrine factors that up-regulate TLR2 to sensitize AVICs for the inflammatory response. We first determined the effect of denature of Pam3 CM (in boiling water for 30 minutes) on its potency to induce AVIC inflammatory response. As shown in **Figure 4 and Figure S2**, denatured Pam3 CM failed to induce the expression of ICAM-1 and VCAM-1 in AVICs. This suggests that heat sensitive factors, likely proteins, from activated monocytes are required to induce the inflammatory response by Pam3 CM.

**Figure 5:**
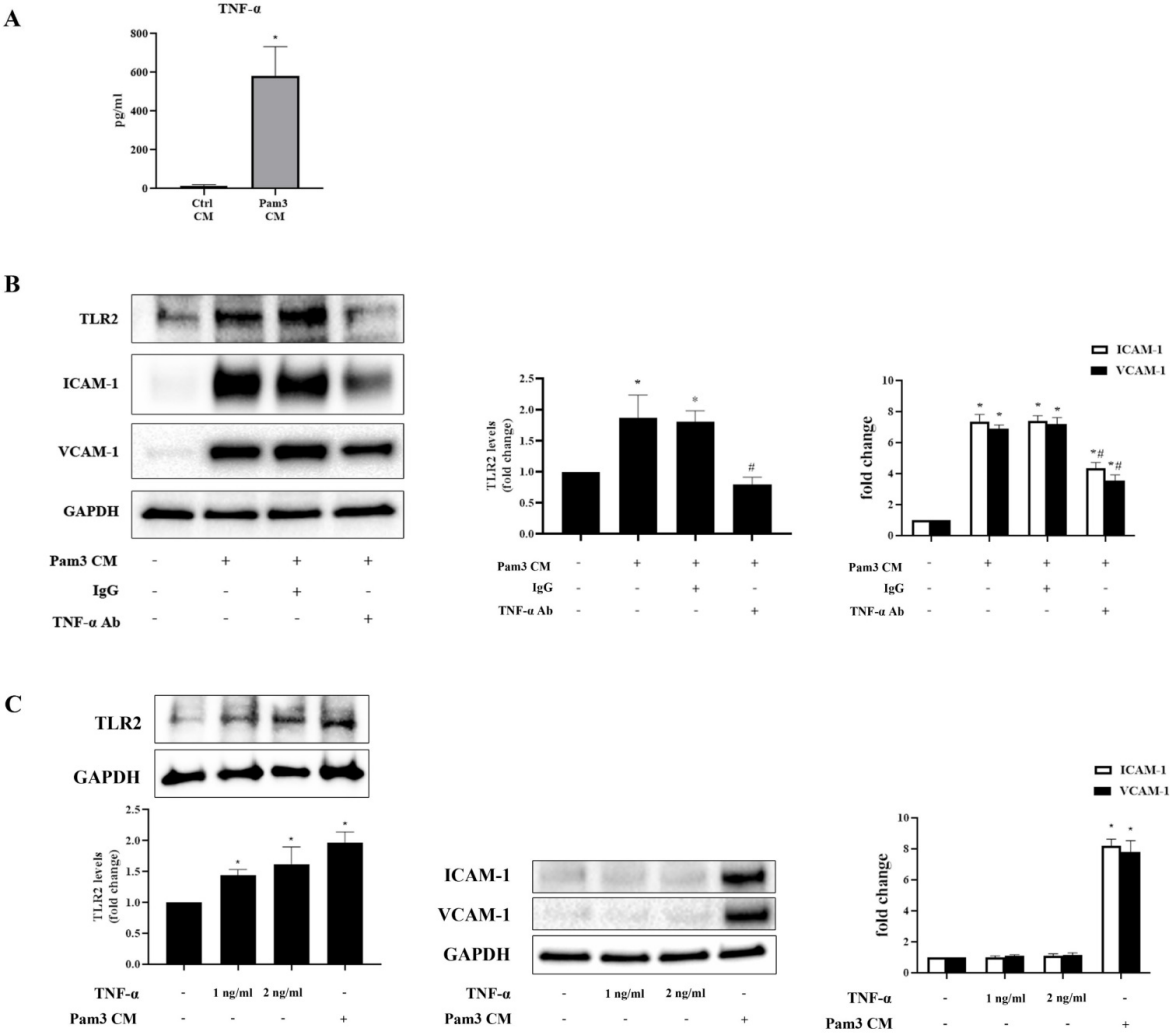
** TNF-α up-regulates TLR2 and contributes to the mechanism underlying the inflammatory responses in AVICs, but recombinant TNF-α is inadequate to induce the inflammatory responses. A.** ELISA assay confirmed that Pam3 CM has greater levels of TNF-α in comparison to control CM. Data are presented as mean ± SEM. n=5, *P<0.05 vs. control CM. **B.** AVICs were treated for 24 hours with Pam3 CM pre-incubated with TNF-α neutralizing antibody (10 µg/ml) or non-immune mouse IgG (10 µg/ml). Neutralization of TNF-α abolished the effect of Pam3 CM on TLR2 up-regulation and attenuated the capacity of Pam3 CM to induce ICAM-1 and VCAM-1 expression in AVICs. *P<0.05 vs. untreated control, *#P*<0.05 vs. Pam3 CM and Pam3 CM + IgG. **C.** AVICs were treated with recombinant TNF-α (1.0 or 2.0 ng/ml) or Pam3 CM for 24 hours. TNF-α in the tested doses increased TLR2 levels in AVICs, but had no effect on the expression of ICAM-1 and VCAM-1. Data are presented as mean ± SEM. n=4 cell isolates from distinct donor valves in each group. *P<0.05 vs. untreated control.

Multiplex-ELISA analysis revealed markedly increased levels of chemokines (MCP-1, IL-8, MIP-1α and RANTES) and cytokine (TNF-α) in Pam3 CM compared to control CM (**Table 1**). As chemokines mainly mediate immune cell migration 29, we hypothesized that pro-inflammatory cytokine TNF-α mediates the up-regulation of TLR2 to sensitize AVICs. We first confirmed high levels of TNF-α in Pam3 CM using ELISA assay (**Figure 5A**). Then, we determined effect of neutralizing TNF-α in the Pam3 CM on AVIC TLR2 levels and expression of ICAM-1 and VCAM-1. As shown in **Figure 5B**, neutralization of TNF-α with a specific antibody abolished Pam3 CM-induced TLR2 up-regulation and markedly attenuated the effect of Pam3 CM on AVIC expression of the adhesion molecules. To determine whether TNF-α in the concentrations observed in Pam3 CM has a pro-inflammatory effect on AVICs, we applied recombinant TNF-α to treat AVICs. As shown in **Figure 5C**, recombinant TNF-α in a concentration of 1.0 or 2.0 ng/ml up-regulated TLR2 levels in AVICs but failed to induce the overexpression of ICAM-1 and VCAM-1. Together, the results show that pro-inflammatory cytokine TNF-α plays a role in the mechanism by which activated monocytes up-regulate TLR2 in AVICs to enhance cellular inflammatory response to TLR2 stimulation.

## Discussion

Aortic valve inflammation promotes valvular tissue fibrosis and calcification associated with CAVD progression. While monocytes/macrophages and AVICs are involved in valvular inflammation in CAVD, the interaction of monocytes/macrophages with AVICs in valvular inflammation is unclear. In this study, we found that: (1) activated monocytes sensitize AVICs for inflammatory response to TLR2 stimulation, (2) monocytes sensitize AVICs by up-regulation of TLR2 through a paracrine mechanism and (3) TNF-α secreted by activated monocytes is responsible for up-regulation of TLR2 and thereby enhances the inflammatory response in AVICs. The results of this study demonstrate a pro-inflammatory interplay between monocytes and AVICs and indicate a novel mechanism by which infiltrated monocytes contribute to CAVD progression.

### Monocytes sensitize AVICs for inflammatory response to TLR2 stimulation by a paracrine mechanism

Growing numbers of studies demonstrate that endogenous factors can function as danger-associated molecular patterns to activate TLRs. Our previous study revealed that neutralization of TLR2 reduces the pro-inflammatory and pro-osteogenic effects of oxLDL in human AVICs 9. Further, we found that several ECM proteins, such as biglycan and matrilin-2, induce inflammatory and osteogenic responses in human AVICs via TLR2 and TLR4 11, 30. The mechanistic role for TLR2 in elevating AVIC inflammatory and osteogenic activities is well established 10, 30. In a previous study, we observed that specific TLR2 activator Pam3CSK4 at a dose of 0.1 μg/ml induces high levels of inflammatory response in human AVICs characterized as the expression of adhesion molecule ICAM-1 31. In the present study, we determined whether monocytes augment AVIC inflammatory response to TLR2 stimulation. We applied a lower dose of Pam3CSK4 (0.03 μg/ml) to stimulate human AVICs, human monocytes and co-culture of AVICs and monocytes. This low dose of Pam3CSK4 failed to induce inflammatory response in AVICs while it induced ICAM-1 expression and MCP-1 production in monocytes, indicating that AVICs are relatively less sensitive to a low level of TLR2 activator in the environment. Streptococcus pneumoniae, a known TLR2 activator, has been reported to upregulate ICAM-1 levels in monocytes 32. Stimulation of TLR2 leads to the activation of NF-κB mainly through the MyD88 signaling pathway. It is likely that the MyD88/ NF-κB signaling pathway play a role in mediating ICAM-1 expression in monocytes exposed to a low dose Pam3CSK4.

Interestingly, low dose TLR2 activator induced massive expression of ICAM-1 and VCAM-1, and marked production of MCP-1 in the co-culture, and immunofluorescence staining of ICAM-1 localized this adhesion molecule in both monocytes and AVICs. Thus, monocytes not only express inflammatory mediator (ICAM-1) in response to a low level of TLR2 activator, but also influence AVICs to execute an inflammatory response to a low level of TLR2 activator. While a synergistic outcome may indicate mutual influences between the two cell types, it is more likely that monocytes somehow interact with AVICs to sensitize them for responding to an otherwise sub-threshold level of TLR2 activator. This notion is supported by the observation that AVICs become responsive to a low level of TLR2 activator for ICAM-1 expression when monocytes are present.

Monocytes interact with neighboring cells through paracrine and juxtacrine signaling 33. Paracrine signaling involves secretion and diffusion of factors over a short distance to induce changes in nearby cells 34. Juxtacrine signaling is dependent upon interaction with adjacent cells through physical contact at the cell surface 35. The sensitization observed in this study appears not require a physical interaction of monocytes with AVICs since conditioned medium, which retains the TLR2 activator and factors secreted by activated monocytes, also up-regulates the levels of ICAM-1, VCAM-1 and MCP-1 in AVICs. Noticeably, the conditioned medium is as potent as activated monocytes in sensitizing AVICs in co-culture for TLR2-induced expression of these two adhesion molecules. Thus, activated monocytes sensitize AVICs to a low level of TLR2 activator mainly though a paracrine mechanism. Indeed, inhibition of TLR2 with a specific inhibitor essentially abrogates the effect of conditioned medium on the expression of ICAM-1 and VCAM-1 in AVICs. Together, the results demonstrate that soluble factors from activated monocytes enhance AVIC sensitivity to TLR2 stimulation by a sub-threshold level of activator. Therefore, subsequent experiments in this study utilized Pam3CSK4-containing condition media diluted in AVIC cultures to a final Pam3CSK4 concentration of 0.03 μg/ml.

In the present study, we observed that low dose of Pam3CSK4 (0.03 μg/ml) increased the expression of adhesion molecules in AVICs co-cultured with monocytes, but not in AVIC culture. As the results of experiments using conditioned medium show that a paracrine mechanism sensitizes AVICs to execute the inflammatory response to a low level of TLR2 activator it is likely that soluble factors released by activated monocytes exert such an effect on AVICs through up-regulation of cellular TLR2 levels, modification of TLR2 distribution or enhancement of TLR2 signaling.

### Up-regulation of TLR2 by a TNF-α-dependent mechanism is involved in sensitization of AVICs for inflammatory response to TLR2 activator

Up-regulation of TLR2 by GM-CSF in neutrophils has been shown to enhance their inflammatory response to TLR2 agonist 36. Our previous study found higher TLR2 levels in AVICs from aortic valves affected by CAVD versus those in AVICs from normal aortic valves, indicating a mechanistic role of TLR2 over-expression in mediating the elevated inflammatory and osteogenic activities in diseased aortic valves 10. In the present study, we found that activated monocytes up-regulate TLR2 expression in AVICs through paracrine signaling and that elevated levels of TLR2 in AVICs correlate to greater sensitivity to a low level of TLR2 activator in the Pam3 CM. Further, prevention of the TLR2 up-regulation in AVICs using a knockdown approach markedly attenuated Pam3 CM-induced inflammatory response. This suggests that up-regulation of TLR2 is an important mechanism by which activated monocytes sensitize AVICs for the inflammatory response to a low level of TLR2 activator that would be inadequate to elaborate AVIC inflammatory responses in AVIC culture.

Activated monocytes are known to produce multiple pro-inflammatory mediators 37. In the present study, multiplex-ELISA identified several pro-inflammatory cytokines/chemokines released by monocytes exposed to a TLR2 agonist. Of all pro-inflammatory cytokines present in Pam3 CM, increased TNF-α levels is outstanding. Neutralization of TNF-α in Pam3 CM abolished the up-regulation of TLR2 levels in AVICs and resulted in significant reduction of inflammatory response to Pam3 CM. These results suggest that TNF-α released by activated monocytes plays a major role in up-regulating TLR2 expression to sensitize AVICs for inflammatory response. To evaluate the effect of TNF-α on TLR2 levels and pro-inflammatory activity in AVICs, we treated AVICs with recombinant TNF-α for 24 hours. The results confirmed that TNF-α at 1.0-2.0 ng/ml, comparable to the concentration in Pam3 CM, up-regulates TLR2 levels, but fails to induce the expression of ICAM-1 and VCAM-1 in human AVICs. It appears that a low concentration of soluble TNF-α is not adequate to induce AVIC inflammatory responses. Exosomes can deliver TNF-α to target cells 38. Exosomal TNF-α released from monocytes may be involved in up-regulation of the expression of adhesion molecules in human AVICs. It is also possible that another factor up-regulated by CM in human AVICs, likely the p55 TNF receptor (TNF receptor 1, TNFR1), is needed to enhance the effect of TNF on adhesion molecule expression. Ligand binding to TNFR1 leads to the activation of NF-κB 39. NF-κB is one of critical transcription factors that regulate TLR expression 40. Nevertheless, the present study shows that TNF-α is capable of up-regulating TLR2 in human AVICs. However, up-regulation of TLR2 is not unique for TNF-α, since multiple pro-inflammatory cytokines including IFNγ, IL-1α and IL-1β have been shown to up-regulate TLR2 expression in various cell types 36, 41-44. Multiplex-ELISA also identified these cytokines in the conditioned medium, but there was no significant difference between control CM and Pam3 CM. Nevertheless, the finding that TNF-α in a concentration close to that in Pam3 CM elevates TLR2 level without inducing the inflammatory response in AVICs supports the notion that TNF-α up-regulates TLR2 expression to sensitize AVICs for inflammatory response to a low level of TLR2 activator.

## Limitations

There are several limitations in this study. First, the sample size is small. While all experiments were repeated using cell isolates from different donors to ensure the reproducibility of the observations, this small group of normal aortic valves used cannot provide information regarding the influence of clinical factors, such as age, gender and heart disease, on AVIC response to the tested stimuli. Second, we focused only on the expression of ICAM-1 and VCAM-1, although high concentrations of TNF-α are capable of inducing the expression of osteogenic mediators 45, 46. The potential effect of monocytes-conditioned medium and TNF-α on the expression of osteogenic mediators, such as Runx and alkaline phosphatase need to be evaluated in future studies. It is also noteworthy that neutralization of TNF-α in Pam3 CM did not completely abolish the inflammatory response in AVICs. This suggests that other pro-inflammatory mediators, in addition to TNF-α, in the conditioned medium may be involved in inducing the inflammatory response in AVICs. Although several pro-inflammatory cytokines were included in the multiplex-ELISA analysis, their levels were not increased in the Pam3 CM. Further work is needed to investigate the TNF-α-independent pro-inflammatory effect of the Pam3 CM.

## Conclusion

**Figure 6:**
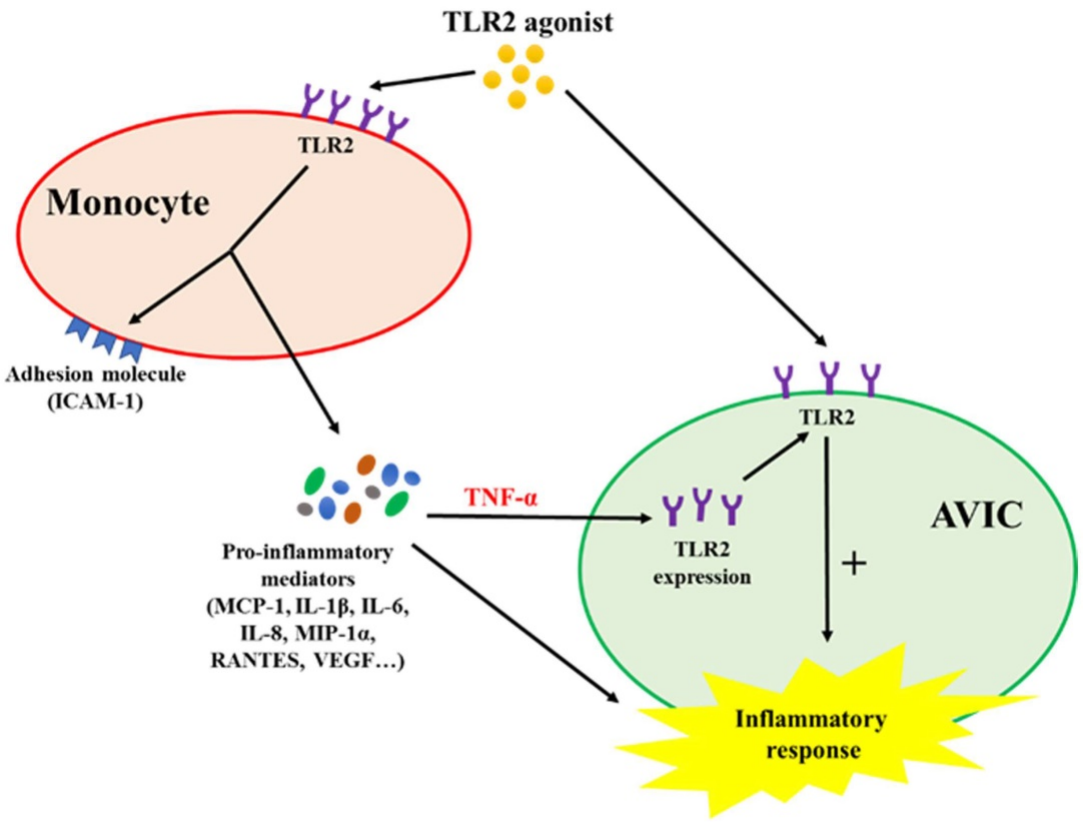
** Schematic diagram depicting the mechanism underlying the interaction between monocytes and AVICs.** Monocytes activated by TLR2 agonist release pro-inflammatory cytokines. Among these cytokines, TNF-α is responsible for up-regulating AVIC expression of TLR2. AVICs with greater levels of TLR2 become hypersensitive to TLR2 stimulation by a low level of activator.

This study demonstrates that activated monocytes use paracrine signaling to sensitize AVICs for inflammatory response to a low level of TLR2 activator. The mechanism of sensitization involves up-regulation of AVIC TLR2 levels by TNF-α secreted from activated monocytes. Infiltrated monocytes in aortic valve tissue may contribute to valvular inflammation associated with CAVD progression by rendering AVICs hypersensitive to TLR2 activators (**Figure 6**).

## Supplementary Material

Supplementary figures and tables.

## Figures and Tables

**Table 1:** Pam3 CM has greater levels of pro-inflammatory mediators

Mediators (pg/ml)	Control CM	Pam3 CM
BMP-2	3.10 ± 0.01	3.41 ± 0.25
BMP-7	0.47 ± 0.30	0.59 ± 0.41
MCP-1	19.8 ± 1.12	2001 ± 129*
G-CSF	1.49 ± 0.89	0.04 ± 0.01
GM-CSF	8.13 ± 2.71	7.45 ± 0.37
IFNγ	341 ± 44.5	359 ± 33.5
IL-11	7.40 ± 0.26	10.7 ± 3.07
IL-17	5.35 ± 1.72	6.28 ± 1.57
IL-1α	16.4 ± 2.52	17.2 ± 2.77
IL-1β	0.75 ± 0.31	2.36 ± 0.99
IL-6	35.3 ± 2.50	87.3 ± 19.3
IL-8	37.5 ± 5.59	3640 ± 286*
MIP-1α	1.06 ± 0.64	3976 ± 210*
RANTES	95.5 ± 0.15	1022 ± 38.0*
TGF-β1	465 ± 60.2	411 ± 64.9
TNF-α	62.4 ± 13.6	507 ± 83.0*
RANKL	12.4 ± 5.46	3.15 ± 1.61
VEGF	226 ± 34.2	345 ± 46.8

Multiplex ELISA assay revealed significantly higher levels of pro-inflammatory cytokine TNF-α and 4 chemoattractants (MCP-1, IL-8, MIP-1α and RANTES) in Pam3 CM compared to control CM. Data are presented as mean ± SEM. n=4, *P<0.05 vs. control CM.

## Abbreviations

CAVD: calcific aortic valve disease
AVIC: aortic valve interstitial cell
TLR: Toll-like receptor
CM: conditioned medium
Pam3 CM: Pam3CSK4 conditioned medium
control CM: control conditioned medium
TNF-α: tumor necrosis factor-α
Pam3CSK4: Pam3CysSerLys4
ICAM-1: intercellular adhesion molecule 1
VCAM-1: vascular cell adhesion protein 1
GAPDH: glyceraldehyde 3-phosphate dehydrogenase
DAPI: 4′,6-diamidino-2-phenylindole
shRNA: small hairpin RNA
MyD88: Myeloid differentiation primary response 88
DMSO: Dimethyl sulfoxide
CU CPT 22: 3,4,6-trihydroxy-2-methoxy-5-oxo-5H-benzocycloheptene-8-carboxylic acid hexyl ester
BMP-2: bone morphogenetic protein 2
BMP-7: bone morphogenetic protein 7
MCP-1: monocyte chemoattractant protein-1
G-CSF: granulocyte-colony stimulating factor
GM-CSF: granulocyte-macrophage colony-stimulating factor
IFNγ: Interferon-γ
IL-11: interleukin 11
IL-17: interleukin 17
IL-1α: interleukin 1α
IL-1β: interleukin 1β
IL-6: interleukin 6
IL-8: interleukin 8
MIP-1α: macrophage inflammatory protein 1-α
RANTES: regulated on activation, normal T cell expressed and secreted
TGF-β1: transforming growth factor beta 1
RANKL: receptor activator of nuclear factor kappa-Β ligand
VEGF: vascular endothelial growth factor
TNF-α Ab: tumor necrosis factor-α neutralizing antibody
Runx: runt-related transcription factor
ELISA: enzyme-linked immunosorbent assay
