# Supplementary material for: Monocytes enhance the inflammatory response to TLR2 stimulation in aortic valve interstitial cells through paracrine up-regulation of TLR2 level
Source: Int J Biol Sci. 2020 Oct 3;16(15):3062–74. doi: 10.7150/ijbs.49332 (PMC7545700; doi:10.7150/ijbs.49332)
Supplement: Supplementary file 1 — Supplementary figures and tables. [file ijbsv16p3062s1.pdf]

Supplemental Data

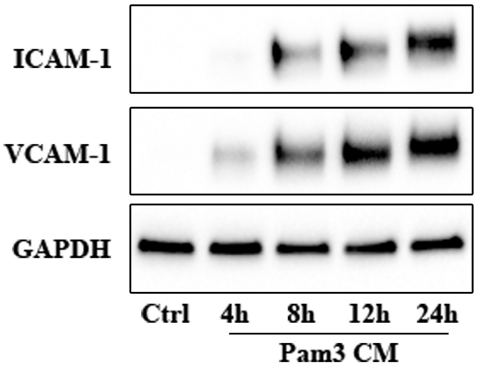

Supplemental Figure 1. Pam3 CM up-regulates ICAM-1 and VCAM-1 levels in AVICs in a time-dependent fashion. AVICs were treated with Pam3 CM for 4 to 24 hours. Representative immunoblot of 3 separate experiments shows that Pam3 CM time-dependently induced ICAM-1 and VCAM-1 expression in AVICs.

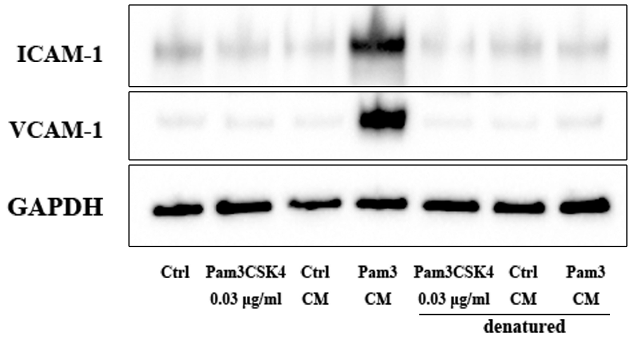

Supplemental Figure 2. The effect of heat-denatured and non-denatured Pam3 CM on AVIC expression of ICAM-1 and VCAM-1. AVICs were treated with boiled or non-boiled solutions Pam3CSK4, control CM and Pam3 CM for 24 hours. Representative immunoblot of 2 separate experiments shows that heat-denatured Pam3 CM failed to induce ICAM-1 and VCAM-1 expression in AVICs.
